# Identification of p63^+^ keratinocyte progenitor cells in circulation and their matrix-directed differentiation to epithelial cells

**DOI:** 10.1186/scrt186

**Published:** 2013-04-11

**Authors:** Renjith P Nair, Lissy K Krishnan

**Affiliations:** 1 Thrombosis Research Unit, Biomedical Technology Wing, Sree Chitra Tirunal Institute for Medical Sciences and Technology, Trivandrum, 695012, India

**Keywords:** Keratinocyte progenitor cells, p63, Cytokeratins, Peripheral blood mononuclear cells, Epithelial cells, Fibrin, Matrix-directed differentiation, Skin-tissue engineering

## Abstract

**Introduction:**

In the event of chronic diabetes or burn wounds, accomplishing skin regeneration is a major concern. Autologous skin grafting is the most effective remedy, but the tissue harvest may create more nonhealing wounds. Currently available skin substitutes have a limited clinical outcome because of immune reactions arising from the xenobiotic scaffold or allogenous cells. Autologous stem cells that can be collected without an additional injury may be a viable option for skin-tissue engineering. Presence of a low number of keratinocyte progenitor cells (KPCs) within the peripheral blood mononuclear cell (PBMNC) population has been indicated. Identification, isolation, expansion, and differentiation of KPCs is necessary before they are considered for skin regeneration, which is the focus of this study.

**Methods:**

Culture of isolated human PBMNCs on a cell-specific matrix was carried out to induce differentiation of KPCs. Flow cytometry and reverse transcriptase polymerase chain reaction were done for epithelial stem cell marker p63 and lineage markers cytokeratin 5 and cytokeratin 14, to track differentiation. Proliferation was confirmed by quantifying the proliferating cell nuclear antigen-expressing cells. Immunostaining with epithelial cell markers, involucrin and filaggrin, was carried out to establish terminal differentiation. Microscopic analysis confirmed growth and survival of KPCs on the dermal fibroblast monolayer and on a transplantable fibrin sheet.

**Results:**

We demonstrated that KPCs are p63^+^ and CD34^-^. The specifically designed composition of the extracellular matrix was found to support selective adhesion, proliferation, and differentiation of p63^+^ KPCs. The PBMNC culture for 12 days under controlled conditions resulted in a homogenous population that expressed cytokeratins, and >90% of the cells were found to proliferate. Subculture for 5 days resulted in expression of filaggrin and involucrin, suggesting terminal differentiation. Transfer of matrix-selected KPCs to a dermal fibroblast monolayer or fibrin supported cell proliferation and showed typical hexagonal morphology of keratinocytes within 15 days.

**Conclusions:**

Circulating KPCs were identified with p63, which differentiated into keratinocytes with expression of the cytokeratins, involucrin and filaggrin. Components of the specifically designed matrix favored KPC attachment, directed differentiation, and may turn out to be a potential vehicle for cell transplantation.

## Introduction

For the cure of chronic ulcers, tissue-engineered skin substitute has been proven to produce a positive outcome. However, because the commercially available skin substitutes use xenobiotic scaffold material or allogenic cells [1], the outcome is limited. Therefore, identification of a suitable cell source and a biocompatible culture matrix is critical for successful skin-tissue engineering.

The major requirements of the cells to be used for skin-tissue engineering are that they should not be terminally differentiated, they must possess proliferation potential, and they must be lineage committed. It has been shown that the bone marrow-derived stem cells (BMDSCs) could differentiate into a number of different cell types, including epidermis [2]. In many instances, BMDSCs leave bone marrow, circulate in the blood, and are found in peripheral blood mononuclear cell (PBMNC) fractions [3]. A subset of PBMNCs is considered to be multipotent and to acquire macrophagic, epithelial, endothelial, neuronal, and hepatocytic phenotypes [4]. The bone marrow-derived mesenchymal stem cells (MSCs) have been shown to differentiate into keratinocyte-like cells *in vitro*[5]. Sasaki *et al.*[6] showed that MSCs are recruited into wounded skin and contribute to wound repair by differentiation into skin cells. Medina *et al.*[7] reported that circulating bone marrow-derived CD34^+^ cells may have the capacity to transdifferentiate into epithelium-like cells. They also showed recently that CD14^+^ monocytes could differentiate into keratinocyte-like epithelial cells [8]. All these results suggests that, other than the basal stem cells that are responsible for normal epithelial homeostasis, circulating progenitors may also contribute to epithelial layer regeneration. However, none of these studies used a specific marker for demonstrating the presence of keratinocyte lineage-committed stem cells in peripheral blood, which could be directed to skin epithelium.

A homologue of p53, p63, is an essential transcription factor for epithelial development and proliferation. This molecule was shown to distinguish keratinocyte stem cells from their transiently amplifying (TA) progenitors involved in skin generation [9]. It was also shown that TA cell cultures express a cell proliferation-associated nuclear antigen (PCNA). Therefore, the presence of p63 indicates that the cells can proliferate, form clones, and maintain stemness. The transcription factor p63 is also required for epidermal lineage commitment, epidermal differentiation, cell adhesion, and basement membrane formation [10]. Once the basal stem cells reach the suprabasal region, p63 may be downregulated; subsequently proliferation may be arrested. Therefore, it is a probable candidate to be present on lineage-committed keratinocyte progenitor cells (KPCs) as well. Because KPCs are present in low numbers in circulation, it is essential for them to have good proliferation potential if they are to be involved in epithelial regeneration. In addition to p63, the stem cells in the basal layer also contain keratin bundles such as cytokeratin 5 (CK5) and cytokeratin 14 (CK14), and once they differentiate, additional molecules such as filaggrin and involucrin are expressed.

Attachment and multiplication of the identified stem or progenitor cell *in vitro* and *in vivo* require an appropriate substrate that has adhesion sites and signaling molecules. In physiology, p63^+^ stem cell proliferation takes place on the basement membrane, which consists of fibronectin, laminin, collagen, and growth factors. Fibrin clot is the natural scaffold that promotes wound healing in almost all regions of the human body and more specifically in skin repair. A naturally formed fibrin clot at the injury site contains important adhesive proteins, such as fibronectin, and growth factors released from platelets and from other cells such as fibroblasts. Exogenous fibrin matrix, carefully composed of cell-specific growth factors, is proven to be suitable for differentiation of endothelial progenitor cell [11] and neural progenitors [12]. Therefore, a fibrin-based, cell-specific matrix composition may be designed to support attachment and differentiation of KPCs. The extracellular matrix (ECM) molecules, which are important for growth of skin cells, are epidermal growth factor (EGF), angiogenic growth factors (AGFs), hyaluronic acid (HA), gelatin, laminin, and fibronectin.

In this study, p63, cytokeratins (CK5 and CK14), involucrin, and filaggrin antigens were used as markers to establish the lineage of KPCs and their differentiation after culture on the specifically designed matrix. The results may lead to a novel approach for treating chronic wounds with a tissue-engineered skin substitute derived from autologous keratinocyte progenitors isolated from blood and grown on a human plasma-derived fibrin matrix.

## Methods

Gelatin, formaldehyde, Histopaque 1077, ascorbic acid, heparin, insulin, 4′,6-diamidino-2-phenylindole (DAPI), hydrocortisone, agarose, and TritonX-100 were from Sigma Aldrich (St. Louis, MO, USA). DMEM: F12 medium, trypsin-EDTA, and antibiotic-antimycotic were from GIBCO BRL (Now part of Invitrogen Corporation, Grand Island NY, USA). Epidermal growth factor (EGF) was from R &D Systems (Minneapolis, MN, USA), and polystyrene culture dishes were from Nunc (Roskilder, Denmark). In-house prepared materials used were bovine hypothalamus extract (BHE) prepared from young calf tissue, according to the method of Maciag *et al.*[13], hyaluronic acid (HA) prepared from human umbilical cord [14] and dialyzed newborn calf serum, cryoprecipitated fibrinogen concentrate from human plasma, and purified human thrombin [15]. The complete medium contained 10% newborn calf serum, 5 μg/ml insulin, 0.3 mM ascorbic acid, 25 μg/ml BHE, 0.5 μg/ml hydrocortisone, and 20 ng/ml EGF, and 10 μl/ml antibiotic-antimycotic. Anti-human p63 antibody (Nova Castra, Newcastle upon Tyne, UK), phycoerythrin (PE) conjugated anti-human cytokeratin5 (Santa Cruz Biotechnology, Santa Cruz, CA, USA), anti-human cytokeratin14 (Nova Castra), FITC-conjugated antihuman proliferating cell nuclear antigen (PCNA) (Caltag, now part of Invitrogen Corporation, Grand Island NY, USA), antihuman involucrin (Nova Castra), and antihuman filaggrin (Santa Cruz Biotechnology) were used as markers for various experiments. Secondary antibodies used were FITC- or PE-conjugated anti-mouse IgG from Santa Cruz Biotechnology or Abcam (Cambridge Science Park, Cambridge, UK).

### Matrix preparation

Culture plates were coated with fibrin composite, according to the established procedure of Chennazhy and Krishnan [15], with a modified fibrinogen cocktail (FC). The modified FC consists of fibrinogen (2 mg/ml), fibronectin (200 μg/ml), gelatin (0.2%), BHE (25 μg/ml), EGF (20 ng/ml), and HA (0.1 mg/ml). The fibrin network in the culture dish were lyophilized and stored at 4°C until use. A fibrin sheet was prepared, as described earlier [16]. In brief, 1.0 ml of fibrin composite, as described earlier, was mixed with 1.0 ml (5 IU) thrombin to make a clot in a culture well (about 3.3 cm diameter) and was lyophilized to form a stable sheet. The fibrin sheet was suspended in the medium for 5 to 10 minutes before using for cell culture.

### Isolation of peripheral blood mononuclear cells

Isolation of PBMNCs from the buffy coat (discards from Blood Bank collected with informed consent) was done with Histopaque-1077 density gradient centrifugation. As per the standard operating procedure of Institutional Ethics Committee, Sree Chitra Tirunal Institute for Medical Sciences and Technology (IEC-SCTIMST), the collection of discarded buffy coat is exempted from review if the donor identity is not publicized. In brief, red blood cells (RBCs) were settled by centrifugation of the buffy coat at 1,200 *g* for 15 minutes in 15-ml centrifuge tubes (Hareus Stratos, Hanau, Germany). Superficial plasma was discarded; PBMNCs at the interface were collected and diluted with equal volumes of Hank Balanced Salt Solution (HBSS), layered over Histopaque-1077 in centrifuge tubes (Nunc, Roskilder, Denmark), and centrifuged at 400 *g* for 30 minutes at 25°C. The layer containing PBMNCs was carefully separated from the plasma-Histopaque interface and was washed with serum-free DMEM: F12 by centrifugation at 150 *g* at 4°C for 10 minutes. The washed PBMNCs were suspended in complete medium.

### Cell culture

The PBMNCs suspended in complete medium were initially seeded in bare 20-cm^2^ culture dishes and were incubated for 1 hour in a humidified incubator under 5% CO_2_ at 37°C. After 1 hour, the medium was removed gently, and fresh medium was added. After 48 hours, the medium was removed, and nonadherent but settled cells were flushed with complete keratinocyte culture medium, and the cells attached to the bare polystyrene surface were discarded. The plastic-nonadherent cells were seeded onto matrix-coated dishes and were incubated in a humidified incubator under 5% CO_2_ at 37°C. The medium was replaced with fresh medium each day until 72 hours; afterward, the medium was changed on alternate days. The morphology of cultured cells was observed with a phase-contrast microscope (Leica Microsystems, DMIRB Wetzlar, Germany).

Fibroblasts were isolated from human foreskin from a local hospital (collected with informed consent) by using a standard protocol [17]. Cells were subcultured, and either the third- or the fourth-passage monolayer was used for seeding KPCs. The protocol for co-culture was to seed PBMNCs in an uncoated dish for 48 hours, transfer to a coated dish, and after 4 days on biomimetic matrix, KPCs were flushed out and seeded onto either a fibroblast monolayer or a fibrin sheet. Culture was continued in the same medium composition as described earlier.

### Immunostaining for fluorescence microscopy

Cells cultured on FC-coated 1.75-cm^2^ wells for a specific period of time were rinsed with phosphate-buffered saline (PBS), fixed with 3.7% formaldehyde in PBS, washed with PBS, and permeated with Triton X-100 (0.1%) in PBS. Cells were then incubated with primary antibodies against p63 (1:50), FITC-conjugated cytokeratin 14 antibody (1:50), or with phycoerythrin (PE)-conjugated cytokeratin 5 (1:50), antiinvolucrin (1:100), and antifilaggrin (1:100). Conjugated secondary antibody was used at 1:200 dilution, and cultures were viewed by using a fluorescence microscope (Leica Microsystems, DMIRB, Wetzlar, Germany). Actin staining was done by using phalloidin conjugated with Texas Red (Molecular Probes, now part of Invitrogen Corporation, Grand Island NY, USA), and for nuclear staining of cells, DAPI (Sigma Aldrich) was used.

### Flow cytometry

Cells in culture for 1 hour, 4 days, 8 days, and 12 days were harvested by flushing out or by using the standard trypsinization protocol (0.25% Trypsin-EDTA for 3 minutes), washed with PBS, fixed with 3.7% formaldehyde in PBS for 20 minutes, washed with PBS, and permeated with Triton X-100 (0.1%) in PBS for 5 minutes. The cells were treated with 1% bovine serum albumin (Sigma-Aldrich, St. Louis, MO, USA) in PBS for 30 minutes and incubated with FITC-conjugated antibodies against PCNA (1:100), CK14 (1:200), phycoerythrin-conjugated CK5 (1:100), and nonconjugated p63 (1:100) antibody for 2 hours. The cells incubated with primary p63 antibodies were washed and treated with FITC-conjugated secondary antibody (1:200) for 1 hour. After the staining procedure, the cells were washed and analyzed by using a flow cytometer (FACS ARIA, BD Biosciences, San Jose, CA, USA). The percentage of marker-expressed cells was calculated by using BD FACS Diva software (BD Biosciences, San Jose, CA, USA).

Sorting of CD34^+^ cells from the PBMNCs was done from the enriched Histopaque gradient centrifugation step, seeded on bare polystyrene for at least 1 hour, and the cells harvested from bare polystyrene were stained with CD34 antibodies conjugated with APC (BD Biosciences, Franklin Lakes, NJ, USA) and sorted by using FACS Aria (BD Biosciences). A portion of the sorted cells was used for analysis of purity, and the remaining cells were fixed, permeated, and stained with p63, as described earlier, and analyzed.

### Reverse transcriptase-polymerase chain reaction

For detecting expression of markers before and after culture, RT-PCR analysis was performed. Total RNA was extracted from the cells in culture on day 1 and day 12 by using Trizol reagent (Invitrogen Invitrogen Corporation, Grand Island NY, USA) according to manufacturer’s protocol. RNA, 1 μg, was converted to cDNA by using superscript III reverse transcriptase enzyme (Invitrogen Corporation). The expression of keratinocyte lineage markers, such as *TAp63*, *ΔNp63*, *CK5*, and *CK14*, was analyzed with RT-PCR. For amplification reactions, Master cycler from Eppendorff (Hamburg, Germany) was used for 40 cycles; the annealing temperature for all reactions was 54°C, and the product was analyzed for molecular size by using agarose gel electrophoresis. *GAPDH* was used as a positive control. The primer sequence used for each gene is given in Table 1. For each gene, an assessment of quality was performed by examining PCR melt curves after real-time PCR by using a Chromo4 system (MJ Research, now part of Bio-Rad, Hercules, CA, USA) to ensure specificity.

**Table 1:** Primers used for the polymerase chain reaction analysis

**Gene**	**Forward primer**	**Reverse primer**	**Amplicon size**
*GAPDH*	5^′^-gcttgtcatcaatggaaatccc-3^′^	5^′^-tccacacccatgacgaacatg- 3^′^	210 bp
*TAp63*	5^′^-aagatggtgcgacaaacaag-3^′^	5^′^-agagagcatcgaaggtggag-3^′^	234 bp
*ΔNp63*	5^′^-ggaaaacaatgcccagactc-3^′^	5^′^-gtggaatacgtccaggtggc-3^′^	294 bp
*CK5*	5^′^-cttgtggagtgggtggctat-3^′^	5^′^-ccacttggtgtccagaacct-3^′^	439 bp
*CK14*	5^′^-gaccattgaggacctgagga-3^′^	5^′^-attgatgtcggcttccacac-3^′^	157 bp

## Results

**Figure 1:**
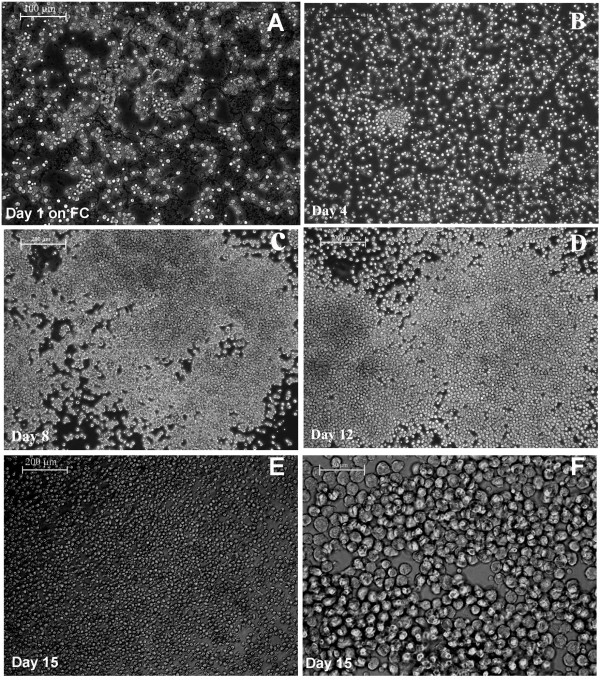
**Representative phase-contrast micrographs of cells on fibrinogen composite (FC) matrix.** (**A**) Cells on the day of seeding; (**B**) on day 4 of seeding; (**C**) on day 8; (**D**) on day 12; (**E, F**), on day 15. A through E are at low magnification (scale bar, 200 μm), and F is at higher magnification (scale bar, 50 μm).

In the preliminary experiments, different compositions of matrices were found to support adhesion of KPCs from PBMNCs with scanty cell survival. For promoting their survival, multiplication, lineage commitment, and differentiation, several modifications were necessary. The final composition of fibrinogen composite (FC) described earlier was found to be suitable for attaining cell survival and multiplication. The cells were initially seeded on bare polystyrene culture wells, and the floating cells were removed at least 3 times within a period of 48 hours of starting the culture. The process involved light swirling of the culture plates and aspiration of medium, which was replaced with fresh medium to eliminate many of the nonspecific cells from the culture. The unattached cells that had settled at the bottom were likely to have many multipotent adult progenitor cells (MAPCs), so they were flushed out and seeded onto the FC-coated dishes. Within 1 hour of seeding on FC, the floating cells were removed. The attached cells were found to form small groups of four to six cells and were uniformly distributed in the culture well (Figure 1A). When the medium was changed on alternate days, more floating cells were removed. Cells were analyzed periodically, and on days 4, 8, and 12, many fields were viewed to check the frequency of cell colonies and morphologic variations, if any, and were micrographed. The size of the colony was found to increase with time (Figure 1B through D). In most of the experiments, the cells grew around the initial colony and filled the culture well in 12 days (Figure 1D). A continuous bed of cells was seen in 15 days (Figure 1E, F). Until the fourth day, cells were not tightly adhered to the culture substrate and could be harvested by flushing with medium, for transfer to other substrates or for analysis. At later periods, cells were attached tightly to the matrix and could be harvested only by the standard trypsinization method.

**Figure 2:**
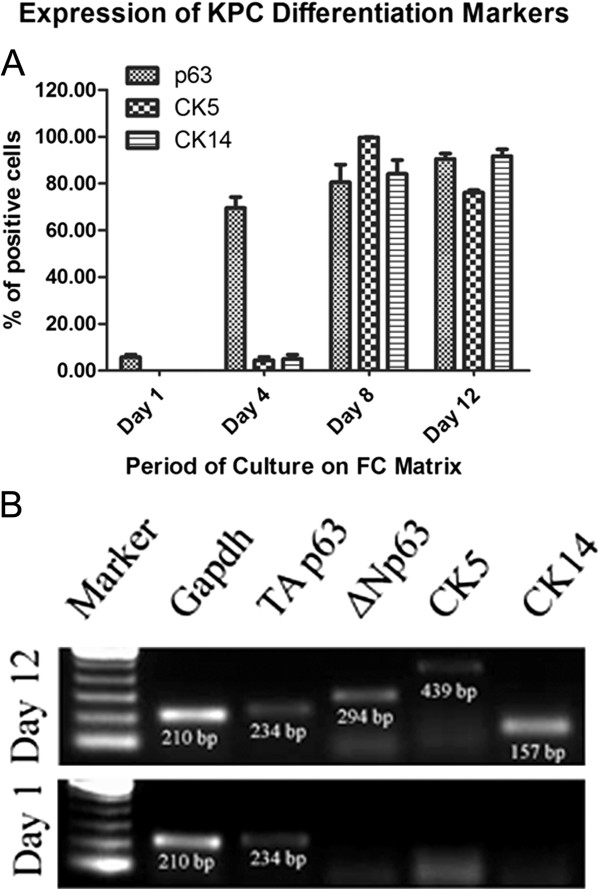
**Evidence for differentiation.** (**A**) Compiled data of flow-cytometric analysis during cell culture. The percentage of cells positive for p63, CK5, and CK14 on each day of analysis during the periods of culture is presented. Periods of analysis against the percentage of positive cells for each parameter are marked in the respective axis. Average ± SD of replicate experiments (*n* = 3), carried out by using different donor PBMNCs are presented. The values are plotted in the bar diagram for comparison of percentage of positive cells on each day. CK14- and CK5-positive cells were absent on day 1. (**B**) Expressions of *p63*, *CK5*, and *CK14* genes on the day of starting the culture and after 12 days of culture. *GAPDH* was the housekeeping gene, and *TAp63* was the only KPC-specific gene that amplified on day 1. Δ*Np63, CK5*, and *CK14* were expressed on day 12, and the products showed specific molecular size.

For identification of KPCs among the cells seeded onto the FC, p63, CK5, and CK14 were analyzed by using flow cytometry. Compiled data of flow-cytometric analysis of cells harvested on days 1, 4, 8, and 12 is shown in Figure 2A. The sample that was analyzed on day 1 was obtained after 1 hour of PBMNC culture on an uncoated dish. On all days, excluding the debris, all cells from the FSC-versus-SSC plot were gated; and in the SSC-versus-FL1/FL2 plot, the gate having <0.5% was selected for detection of the positively stained population. Among the cells obtained after 1 hour, few were p63^+^ in the gated region. The p63^+^ cells were prominent in the monocyte region of the FSC-versus-SSC plot. No CK14^+^ or CK5^+^cells were detectable on day 1 of the culture. Tracking of progenitor /differentiation markers from days 1 to 12 was made possible by using culture of single-donor PBMNCs in at least four culture wells of 10 cm^2^ area, and cells from each well were aspirated for measurement on each specified day (one dish per period). From day 8 onward, almost all cells in the FSC-versus-SSC plots were in a single cluster, which indicated that the cells were homogeneous as compared with previous periods in terms of size and granularity. By day 12, about 90% of cells were positive for both p63 and CK14, but the percentage of CK5 was lowest among the three antigens. Of the cells that survived on day 4, 69% ± 8% were p63^+^, 4% ± 2.5% were CK5^+^, and 4.9% ± 3% were CK14^+.^ This finding suggests that all p63^+^ cells may have survived, and some of them seemed to have differentiated into keratinocyte lineage with CK5 and CK14 expression by day 4. Obviously, specific KPC adhesion to the FC occurred, and floating cells were eliminated from the culture over a period of 4 days. The higher percentage of p63^+^ cells on day 4 as compared with day 1 may be due to the elimination of large number of nonspecific cells from the culture and proliferation of p63^*+*^ KPCs. A remarkable difference was seen in CK5 and CK14 expression on cells cultured for 8 days. Whereas p63^+^ cells were about 80% of the total viable population, >95% were CK5^+^, and >80% were CK14^+^. Average ± standard deviation (SD) of the values from three donors (Figure 2A) show similar progression of expression for p63, CK5, and CK14, with the lowest SD by day 12 of culture. A differentiation experiment was done by using PBMNCs from many other donors for confirming differentiation; however, tracking and quantification of marker expression by using three donors only is presented here. Overall, it was observed that if the PBMNC yield was good, the cell survival and expression of markers was also as good, as the data show in Figure 2. But if PBMNC yield and seeding density were poor, cell survival and marker expression were poor, too. It appeared that seeding density is crucial for KPC survival, with the best results when the seeding density of leukocytes was about 1 × 10^6^/cm^2^ of the biomimetic matrix.

In Figure 2B, results of the gene-expression analysis are shown. In cells on day 1, one isoform of p63 (*TAp63*) was expressed, but none of the other tested markers was amplified in 40 cycles of reaction. In cells on day 12, *TAp63*, *ΔNp63*, *CK5*, and *CK14* genes were clearly expressed. These data confirm that p63, CK5, and CK14 are expressed when PBMNCs were cultured on the KPC-specific niche in this study. How cells were stimulated to the keratinocyte lineage during the later period of cell culture is beyond the scope of this study. It is likely that the molecules present on the culture matrix and in the medium together would have influenced the keratinocyte lineage commitment and expression of more keratinocyte markers.

**Figure 3:**
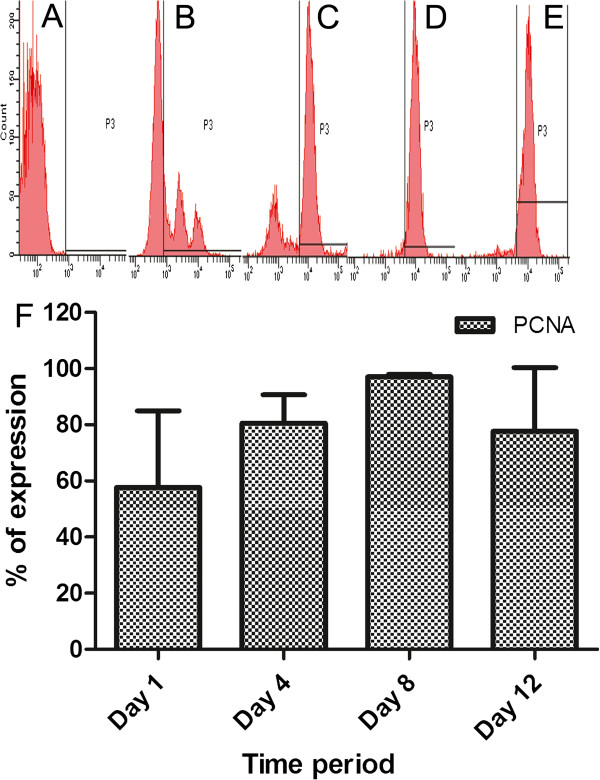
**Data on flow-cytometric analysis of proliferation marker in cells during the period of culture.** PCNA analysis of the cells was done after staining the cells with FITC-conjugated antibodies. (**A**) Representative histogram of unstained control. (**B** through **E**) histograms of PCNA-stained cells on day 1, day 4, day 8, and day 12, respectively, and (**F**) graphic representation of compiled data. Percentage of PCNA-positive cells from three replicate experiments is compiled, and the average ± SD of data from experiments carried out by using different donors’ PBMNCs is presented as a bar graph to compare percentage of PCNA-positive cells between the days of analysis. Days of analysis are marked on the x-axis, and percentage of positive cells on the y-axis of the bar diagram.

The KPCs were found to proliferate during the culture period, and big clusters were found frequently throughout the culture surface. The proliferation marker used was PCNA, and an average of 60% of the gated cells were PCNA positive on day 1; gradually the average reached >95% by day 8, but by day 12, the percentage of proliferating cells decreased to 80% (Figure 3). The reason for the lower percentage of proliferating cells on the initial day may be that the cells were more heterogeneous at that time, and other nonspecific and nondividing cells contaminated the gated region; large donor-to-donor variability was seen during this period. Once they became more homogeneous on day 8 with >95% of cells expressing CK5 (Figure 2A), they were all expressing PCNA as well (Figure 3). The proliferation potential was reduced by day 12, may be because terminal differentiation progressed.

**Figure 4:**
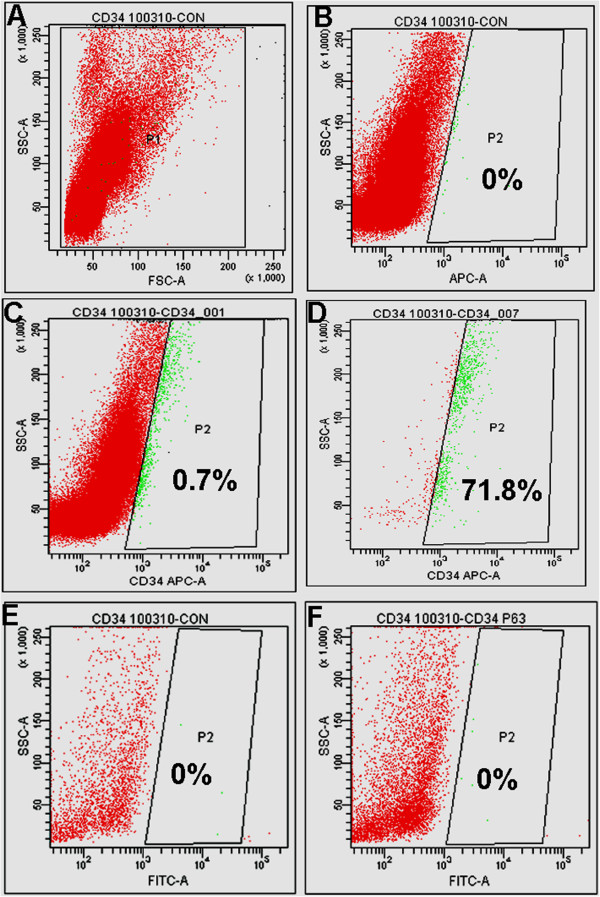
**Data on flow-cytometric analysis of sorted CD34**^**+ **^**cells for the presence of p63.** For analysis of CD34, primary antibodies conjugated with APC (FL1), and for bound p63 antibody, secondary antibodies conjugated with FITC (FL2) were used. (**A**) Representative FSC-versus-SSC plot of unstained PBMNCs flushed from uncoated PS plates. (**B**) Plot of FL1 versus SSC of unstained PBMNCs. (**C**) Representative plot of FL1-CD34-stained PBMNCs before sorting. (**D**) Representative plot of FL1-CD34 sorted cells. (**E**) Representative plot of FL2-versus-SSC sorted cells. (**F**) Representative plot of SSC-versus-FL2-p63 stained cells.

The surface marker CD34 has been reported to be positive on many MAPCs in circulation. To test for its presence on p63^+^ KPCs, CD34^+^ cells were sorted by using FACS Aria. Purity of the sorted cell population was >70%. When the sorted cells were permeated and stained with p63 antibodies, none of the CD34^+^ cells was found to be p63^+^ (Figure 4A through F). Even though KPCs are likely to be of bone-marrow origin, once committed to the lineage and expressed p63, they do not seem to be CD34^+^. The data were consistent in three sorting experiments, which were done by using three different donor PBMNCs.

In this study, we found that p63 is a useful marker to identify keratinocyte progenitors in the circulating blood, which could survive in the culture. With a nuclear antigen, permeation of cells is necessary to stain p63, so it cannot be used as a marker to sort KPCs from PBMNC fractions. Because the cells are CD34^-^, it too cannot be used to sort KPCs from PBMNCs; therefore, matrix-directed cell attachment, proliferation, and differentiation are the most suitable strategies to culture-amplify numbers of KPCs for use in regenerative medicine.

**Figure 5:**
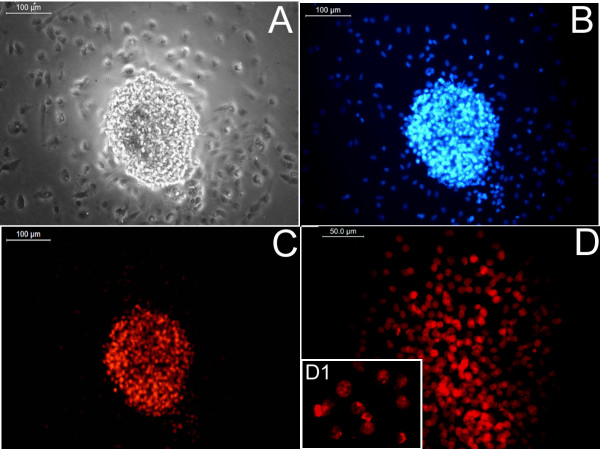
**Identification of p63-specific cells in clusters.** (**A**) Light micrograph of a representative cluster on day 4. (**B**) DAPI-stained cell cluster. (**C**) Immunostained p63-positive cells in the cluster. (**D**) Magnified micrograph of p63^+^ cells. (**A** through **C**) The same cluster is imaged with different filter settings (scale bar, 100 μm). (D) Scale bar, 50 μm; D1 40×.

To confirm keratinocyte lineage commitment and differentiation, cultures were fixed and stained with five markers, which included p63, CK5, CK14, filaggrin, and involucrin. Fibroblasts from human foreskin were used as negative control for all antigens (not shown). Most of the cells in the clusters formed by day 4 of culture (Figure 5A, B) stained positive for p63 (Figure 5C); from the magnified micrograph (Figure 5D, D1), antigen seems to be located in the nucleus. These data correlated well with the flow-cytometry data obtained on day 4 (Figure 2A), which suggests that about 70% of the cells in culture dishes are p63^+^. Some of the cells in the periphery were also p63^+^, but could not be focused together with the cluster when images were taken.

**Figure 6:**
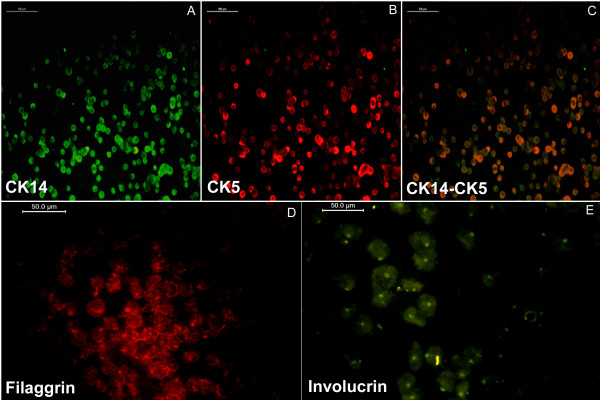
**Immunofluorescence of cells for differentiation markers.** (**A**) Representative image of cell stained with FITC-conjugated CK14 antibody. (**B**) Representative image of PE-conjugated CK5 antibody. (**C**) Merged images of A and B. Both CK14 and CK5 antigens are distinctly located in the cytoplasmic region. (**D**) Representative image of cells stained with anti-involucrin antibody developed by PE-conjugated secondary antibody on day 17. (**E**) Representative image of cells stained with anti-filaggrin antibody developed by using FITC-conjugated secondary antibody on day 17. For negative control, fibroblasts were stained with each antibody (not shown). Scale bar is 100 μm for cytokeratin-stained images and 50 μm for filaggrin- and involucrin-stained images.

For CK5, CK14, filaggrin, and involucrin immunostaining, the cells in first-passage culture were used, and stained cells are shown in Figure 6. After subculture, the KPCs were able to survive when they were seeded onto a fresh matrix-coated dish. Both antigens CK14 (Figure 6A) and CK5 (Figure 6B) stained the cytoplasmic region of the cells, leaving the nuclear region distinctly negative; thus, the flow-cytometry data were substantiated by immunofluorescent micrographs. Merged images of CK14 and CK5 shown in Figure 6C illustrate colocalization of the cytokeratins. The cells expressed involucrin, which appeared as a distinct granule in the cell cytoplasm (Figure 6D). They also showed expression of filaggrin, which appeared as a string of granules in cytoplasm (Figure 6E). The cells did not express involucrin and filaggrin unless they were allowed to grow for 5 days in subculture. Passaging to a fresh matrix was done after 12 days of culture on the initial differentiation matrix.

**Figure 7:**
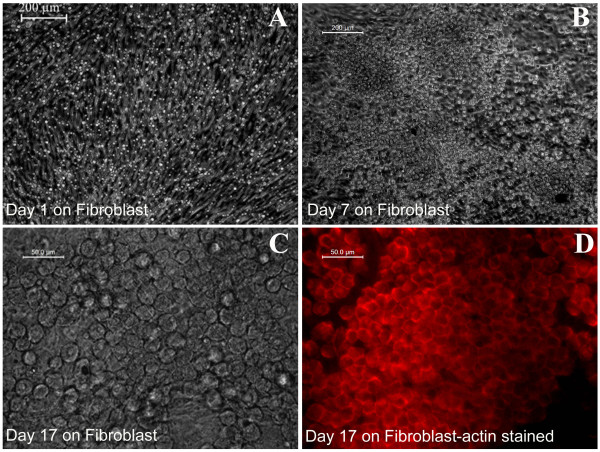
**Representative images of keratinocyte progenitor cells (KPCs) on fibroblast monolayer.** (**A**) Representative image of KPCs on fibroblast monolayer on the day of seeding. (**B**) KPCs on fibroblast on day 7. (**C**) KPCs on fibroblast on day 17. (**D**) Actin-stained KPCs on fibroblast. Magnification is the same for A and B (scale bar, 200 μm); and for C and D, the scale bar is 50 μm.

**Figure 8:**
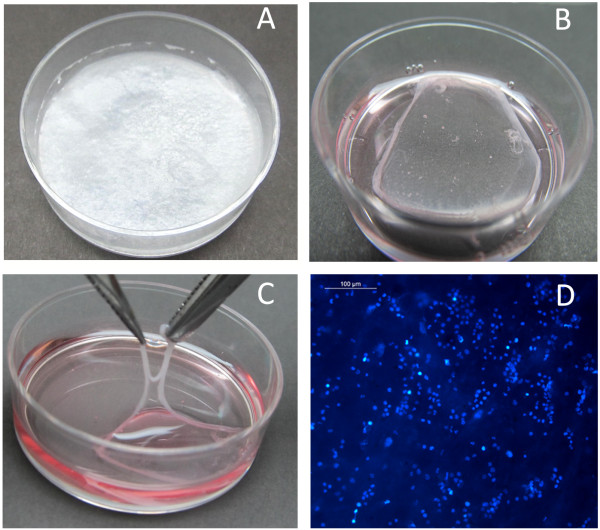
**Fibrin sheet developed for transplantation of keratinocyte progenitor cells (KPCs).** (**A**) Lyophilized fibrin sheet that was cast in culture dish (3.3-cm diameter). (**B**) Fibrin sheet suspended in medium for seeding KPCs. (**C**) Skin-like fibrin sheet after 2 days of KPC culture. (**D**) DAPI-stained KPCs grown on fibrin sheet for 2 days after transferring from FC matrix.

When matrix-selected cells were transferred to the fibroblast monolayer on day 4 of culture, attached cells were distributed throughout the culture area (Figure 7A). After 7 days of growth on the fibroblast surface, clones of KPC could be seen (Figure 7B). By day 17, cells were found to have covered the fibroblast layer, except in some area (Figure 7C). The cells showed the typical hexagonal morphology of keratinocytes on staining them with actin (Figure 7D). A fibrin sheet, which can be used as a biomimetic scaffold to transplant KPC, is shown in Figure 8; a lyophilized sheet is shown in Figure 8A. The scaffold is stable and can be lifted with forceps for implantation, as shown earlier [14], and KPC isolated *in vitro* can be transplanted onto the sheet. To demonstrate that the cells attach and survive on a fibrin sheet after 4 days in the culture dish, cells were flushed out and were transferred to the fibrin sheet suspended in the medium (Figure 8B). Within 2 days of culture, the fibrin scaffold appeared as a thin membrane-like structure that could be lifted (Figure 8C). Handling of the sheet after culture was difficult for imunostaining; so simple DAPI staining was done to demonstrate that the cells transferred from the biomimetric matrix-coated culture dish survived on the fibrin sheet as well. Because of the three-dimensional and porous structure of the sheet, the cells penetrated into the matrix and DAPI-stained nucleus (Figure 8D) and were seen in different planes under the fluorescence microscope. The flow-cytometric analysis had proven that by day 4, >70% of the cells in the fibrin-coated culture dish were p63^+^ (Figure 2A), so the DAPI-stained cells are expected to be p63^+^ KPCs.

Thus when PBMNCs were cultured under the specified conditions on the fibrin matrix, which comprised growth factors, adhesive proteins, and hyaluronic acid, a homogeneous population of KPCs was obtained. The results are promising because KPCs differentiate on the matrix and can be transplanted by using a dermal equivalent such as a fibrin sheet or a fibroblast sheet to support KPC survival and differentiation.

## Discussion

Application of stem cells in skin grafting and tissue engineering is an important area of research in regenerative medicine. An important step in the use of stem cells for tissue engineering is to obtain a relatively pure population that retains multiplication potential. The primary objective of this study was to find an easy-to-access autologous adult stem cell or progenitor cells for skin-tissue engineering. Further, a well-defined and efficient protocol must be developed for directing the commitment and differentiation of stem cells into the keratinocyte lineage for epidermal regeneration. The development of such protocols would reduce the likelihood of spontaneous differentiation of multipotent stem cells into undesired lineages on transplantation.

In this study, we identified p63^+^ KPCs in the circulation by using flow cytometry, but the origin is not clear. They may have reached circulation either from the bone marrow or from the epidermal compartment. Once committed to the specific lineage that expresses p63, they do not seem to be CD34^+^. Use of p63 as a marker for identification of circulating KPCs is a novel approach that we used in this study. To the best of our understanding, this is the first report on the presence of p63^+^ cells in blood. We used a matrix-directed signaling strategy to amplify the number of progenitors and thus generated a homogeneous lineage committed culture that co-expressed p63, CK5, and CK14.

The presence of transit-amplifying cells at the injured tissue has been reported, and they probably originated from bone marrow and differentiated into keratinocytes [18]. They showed that the extent of skin damage influences the engraftment of bone marrow-derived progenitors, and the process continues until 3 weeks after the injury. Korbling and colleagues [19] found that on transplanting peripheral blood stem cells, they differentiated into mature epithelial cells in skin, lungs, gastrointestinal tract, and liver. The results of other studies suggest that adult stem/precursor cells may cross the lineage and germ-layer boundaries and give rise to nonhematopoietic cells. Such transdifferentiation is dependent on the niche to which they home and also on the signals that are received from the local milieu [20,21]. It is likely that circulating progenitors may migrate to damaged areas and participate in the local tissue regeneration [22].

The results of the current study suggest that PBMNCs could be a probable source for isolation and proliferation of KPCs *in vitro* for their potential use in regenerative medicine. It may be relevant in the event that the local milieu is unfavorable for engraftment, mainly due to poor blood circulation at the injured area. Under such circumstances, transplantation of KPCs along with a biomimetic scaffold as cell carrier is likely to enhance epithelialization.

Various studies have identified the roles of p63 in epidermal regeneration, which include lineage commitment, differentiation, cell adhesion, and basement membrane formation. This transcription factor p63 was identified on the basis of its sequence homology with the tumor-suppressor gene *p53*[23]. However, unlike p53, p63 is expressed in a tissue-specific manner and is expressed in stratified epithelia, such as the epidermis. During normal epidermal development, basal stem cells are induced to express epidermal keratins CK5 and CK14 [24,25], and the induction process is dependent on p63. Expression of p63 in cultured cells, single-layered epithelia, or embryonic stem cells also resulted in the expression of CK5 and CK14 [26-29]. Therefore, coexpression of p63, CK5, and CK14 in our experiment is an excellent indication of the epidermal lineage commitment of KPCs.

Our hypothesis was proven true as we found that circulating KPCs express p63. Here, analysis of sorted CD34^+^ cells confirmed that they are p63^-^. We found that after enrichment by Histopaque gradient centrifugation and removal of plastic adherent cells, about 4% of cells are p63^+^; gene amplification was clearly evident. Considering the multipotency of progenitors in PBMNCs, predifferentiated and lineage-committed KPCs may be better suited for regenerative medicine to heal chronic wounds. With progress in the culture period on the niche that was designed during this study, the cells became CK5^+^ and CK14^+^ in flow cytometry, immunofluorescent staining, and RT-PCR. It is interesting to note that the proliferation potential of the cells in culture tends to be reduced by day 12, probably because the lineage commitment progressed. When the cells were subcultured and stained for terminal differentiation markers involucrin and filaggrin, they turned positive for both these antigens. Filaggrin is an intermediate filament (IF)-associated protein that aggregates keratin IFs *in vitro* and is thought to perform a similar function during the terminal differentiation of epidermal keratinocytes [30]. Filaggrin expression resulted in reduced keratinocyte proliferation and caused an alteration in cell-cycle distribution consistent with a post-G_1_-phase arrest. We also found that PCNA expression was reduced by 15 days of KPC culture. In keratinocytes, expression of involucrin may be regulated by cell-substratum contact, and fibronectin at high concentrations can inhibit the expression [31]. In the matrix that we composed, fibronectin content is <2 μg/cm^2^ of the culture surface, and it has not affected involucrin expression on KPCs.

Previously, we reported that the fibrin matrix can be composed with growth factors and glycosaminoglycans for specific requirements such as growth and differentiation of vascular cells and neural cells. Fibrin is abundant, well characterized for its soft elasticity, and is useful as a hemostatic agent and as a scaffold for tissue engineering. Specific binding of fibrin with fibronectin, hyaluronic acid, and many growth factors, as well as clot components, is well established [32]. The bioactivity of RGD sites on fibrin and fibronectin, through which it can interact with integrin, makes it an attractive matrix for stem cell differentiation and tissue engineering. Modulation of its properties has even permitted differentiation of MSCs into osteoblasts and mouse embryonic stem cells to neural and astroglial lineages [33,34].

In this study, we demonstrated that a fibrin substrate composed with specific growth factors and hyaluronic acid permits attachment and growth of circulating keratinocyte progenitors.

Survival of keratinocytes *in vitro* and *in vivo* requires a dermal-equivalent matrix as the niche. When p63^+^ cells were enriched by using a biomimetic matrix-coated dish and transferred to the fibroblast monolayer, KPCs formed colonies and multiplied to cover the fibroblast layer. In skin, stem cells reside on the underlying basement membrane, where they self-renew and form transit-amplifying cells, but they do not anchor tightly to the underlying niche. In the initial period, as no trypsinization is needed to transfer the KPC from the selection niche to the fibroblast sheet or the fibrin sheet, the protocols standardized in this study have potential applications in KPC transplantation and for *in vitro* construction of a skin substitute for epidermal regeneration.

## Conclusion

It is a novel finding that p63 can be used as marker to identify/enumerate KPCs in PBMNCs, which can be collected by using a noninvasive procedure. In 4 days of PBMNC culture on the designed biomimetic niche, KPC was enriched to about 70% homogeneity. Within 12 days of culture, the cells coexpressed p63, CK5, and CK14. The differentiation progressed during subculture and expressed involucrin and filaggrin and showed morphologic features of keratinocytes. After 4 days of PBMNC culture on the biomimetic niche, cells can be easily transferred to a fibrin/fibroblast sheet for continued growth and differentiation. The niche that was standardized for KPC isolation, expansion, differentiation, cell survival, and the protocol for transfer of cells to dermal equivalent may be translated into clinical use for cell/skin-substitute transplantation.

## Abbreviations

AGF: angiogenic growth factor; BHE: bovine hypothalamus extract; BMDSC: bone marrow-derived stem cell; cDNA: complimentary deoxyribonucleic acid; CK14: cytokeratin 14; CK5: cytokeratin 5; CO2: carbon dioxide; DAPI: 4^′^,6-diamidino-2-phenylindole; DMEM: Dulbecco Modified Eagle Medium; ECM: extracellular matrix; EGF: epidermal growth factor; FACS: fluorescence-activated cell sorting; FC: fibrinogen composite; FITC: fluorescein isothiocyanate; HA: hyaluronic acid; HBSS: Hank balanced salt solution; IgG: immunoglobulin G; IU: international unit; KPC: keratinocyte progenitor cell; MAPCs: multipotent adult progenitor cells; MSC: mesenchymal stem cell; PBMNC: peripheral blood mononuclear cell; PBS: phosphate buffered saline; PCNA: proliferating cell nuclear antigen; PE: phycoerythrin; RNA: ribonucleic acid; RT-PCR: reverse transcriptase-polymerase chain reaction; SD: standard deviation; TA: transiently amplifying.

## Competing interests

Authors have no competing interests with regard to publication of this manuscript.

## Authors’ contributions

The first author carried out the experiments and standardized several new protocols, compiled the data, and prepared the draft manuscript. The second author conceived the idea, analyzed the data, and fine-tuned the manuscript. Both authors read and approved the final manuscript.

## Authors’ information

RPN is a graduate student nearly completing PhD research. This work is part of his PhD dissertation. Currently he is evaluating *in vivo* models to test the hypothesis that the niche standardized in this study may be used for cell transplantation.

LKK has been a scientist and leader for many tissue-engineering programs, conceptualized that a fibrin-based cell-specific niche may be used for differentiation of stem cells (MSC) and circulating progenitors to become differentiated endothelial cells, smooth muscle cells, neurons, dermal fibroblasts, keratinocytes, and cardiomyocytes for future applications in regenerative medicine.
